# High‐affinity Bet v 1‐specific secretory IgA antibodies in nasal fluids protect against birch pollen allergy

**DOI:** 10.1111/all.14782

**Published:** 2021-03-04

**Authors:** Sara Huber, Roland Lang, Claudia Asam, Fatima Ferreira, Thomas Hawranek, Gabriele Gadermaier

**Affiliations:** ^1^ Department of Biosciences University of Salzburg Salzburg Austria; ^2^ Department of Dermatology and Allergology University Hospital of the Paracelsus Medical University Salzburg Salzburg Austria

**Keywords:** allergens and epitopes, allergy diagnosis, immunologic tests, mucosal immunity, pollen

To the Editor,

Nasal fluid antibodies act as specific barrier molecules against inhaled antigens through neutralization and shielding mechanisms. They comprise (i) the most abundant and locally produced dimeric secretory IgA (SIgA), (ii) pentameric secretory IgM (SIgM), and (iii) monomeric IgG from passive leakage through blood.^1^ The few allergy studies on nasal fluid antibody subclasses showed divergent results regarding quantification of total or allergen‐specific reactivities.^2^, ^3^, ^4^ So far, comprehensive profiling of antibody subclasses combined with functionality tests is lacking.

In this study, ten non‐allergics (NA) and ten birch pollen allergics (BPA) from Austria were recruited (Ethics Commission Land Salzburg, 01/20/2011). NA were free of allergy symptoms and IgE negative to birch pollen and Bet v 1 (Table 1). BPA suffered from rhinitis/rhino‐conjunctivitis, and two patients also presented with asthma. BPA were SPT‐positive to birch pollen with medium/high serum IgE levels to birch pollen (mean = 21.8 kU/L) and Bet v 1 (mean = 23.2 kU/L) (Figure E1).

**TABLE 1:** Demographic of study participants and responses to birch pollen

Nasal fluid donor	Age [years]	Sex	Birch pollen		Serum ImmunoCAP		
			Allergy symptoms	Skin prick test	Bet v 1‐specific IgE [kU/L]	Birch pollen‐specific IgE [kU/L]	Total IgE [kU/L]
NA 1	29	F	None	np	<0.01	0.02	232
NA 2	28	M	None	np	<0.01	<0.01	3.7
NA 3	33	M	None	np	<0.01	<0.01	63.3
NA 4	36	M	None	np	<0.01	<0.01	45.3
NA 5	22	F	None	np	<0.01	0.07	29.8
NA 6	34	M	None	np	<0.01	<0.01	4.6
NA 7	33	F	None	np	<0.01	0.01	89.7
NA 8	40	M	None	np	<0.01	<0.01	31.4
NA 9	32	M	None	np	<0.01	<0.01	5.9
NA 10	29	F	None	np	<0.01	<0.01	46.1
BPA 1	25	M	R	+++	26.7	24.2	133
BPA 2	28	M	RC	++	0.7	1.3	7.3
BPA 3	51	F	RC	++	7.7	9.3	90.4
BPA 4	23	M	R	++++	16.0	19.9	31.4
BPA 5	60	F	RC	++	14.9	15.2	28.3
BPA 6	49	F	RC	++++	6.2	6.0	28.7
BPA 7	22	M	RC	+++	65.3	58.9	200
BPA 8	41	F	RC, asthma	++	4.5	4.0	125
BPA 9	61	F	RC, asthma	++++	12.8	28.5	206
BPA 10	36	F	RC	+++	57.0	64.5	161

Note

NA 1–10, non‐allergic nasal fluid donors; BPA 1–10, birch pollen‐allergic nasal fluid donors; R, rhinitis; RC, rhino‐conjunctivitis; ++, double positive; +++, triple positive; ++++ fourfold positive; np, not performed.

Using nasal fluids from NA and BPA obtained immediately after the birch pollen season, antibody subclass reactivity to Bet v 1 was determined (Figure 1A). Bet v 1‐specific IgE in nasal fluids of BPA was generally low due to the mild sampling technique, but patients with high serum IgE also showed elevated levels in nasal fluids (Table E1). Interestingly, BPA showed significantly higher nasal fluid IgG4 (*p* < 0.001) and IgG (*p* < 0.01) compared to NA. This observation is a consequence of elevated serum IgG4 that accompanies IgE production in allergics, as nasal fluid IgG is not produced locally but originates from serum transudation.^5^, ^6^ Indeed, Bet v 1‐specifc IgG was also higher in serum of BPAs and correlated well with serum IgG (Table E1). High Bet v 1‐specific SIgA and moderate SIgM reactivity was observed, revealing no difference between NA and BPA (Figure 1A).

**FIGURE 1:**
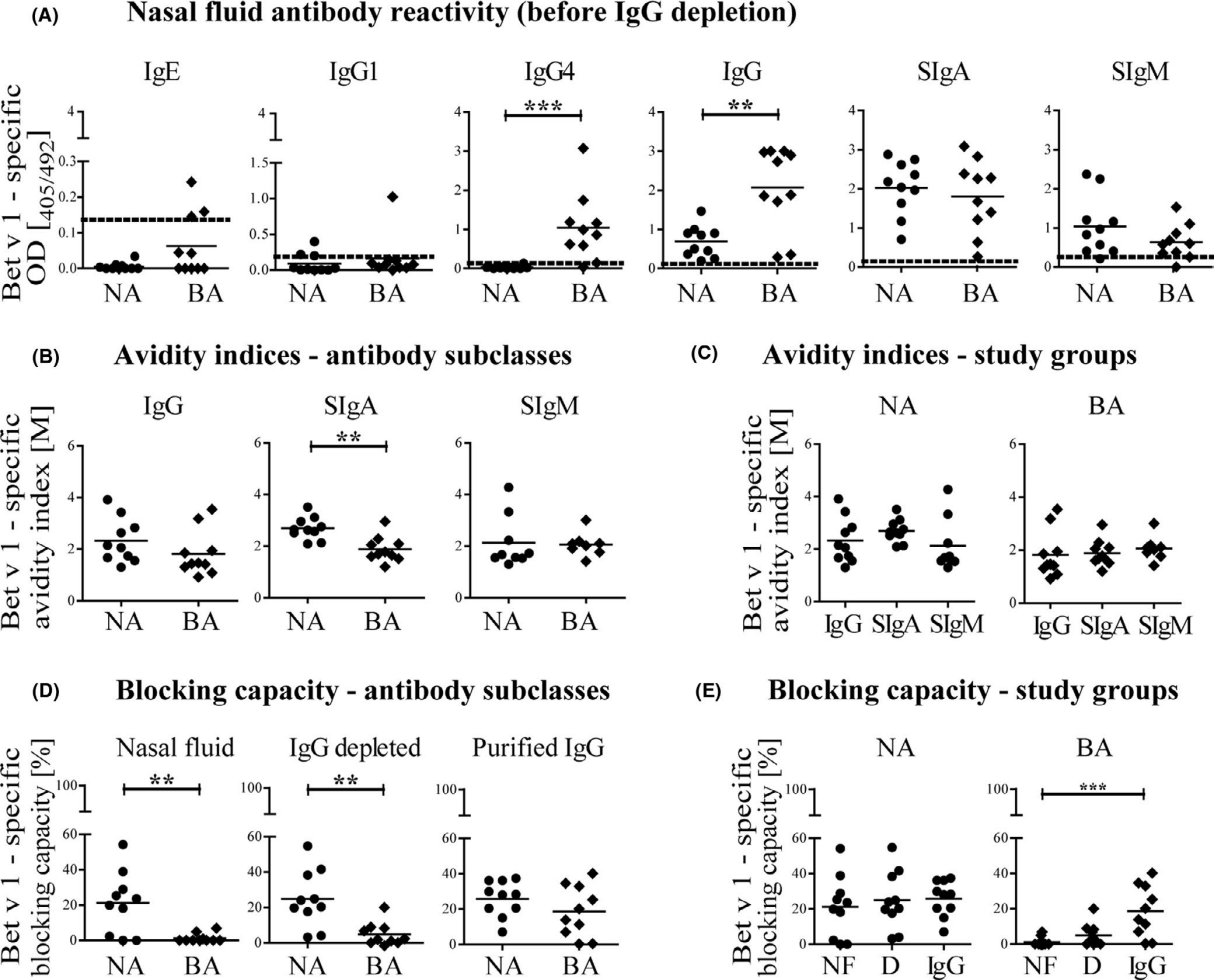
Specific antibody reactivity to Bet v 1 in nasal fluids (NFs) of non‐allergic (NA) and birch pollen allergic (BPA) individuals. (A) Antibody subclass reactivities in NFs analyzed by ELISA. Mean reactivities are indicated by bars, and dotted lines represent LOD (3xSD of buffer control). Statistics using Mann‐Whitney test. (B) Comparison of Bet v 1‐specific antibody avidities between study groups. Statistics using Mann‐Whitney test. (C) Comparison of mucosal antibody avidities within each study group. Statistics using Kruskal‐Wallis and Dunn's post‐test. (D) Percentage blocking capacity of NF and fractions to inhibit serum IgE binding to Bet v 1 compared by antibody subclasses. Statistics using Mann‐Whitney test. (E) Percentage blocking capacity of complete NF (CNF), SIgA/SIgM enriched NF (SIgA/SIgM) and purified IgG (IgG) to inhibit serum IgE binding to Bet v 1 compared by study groups. Statistics using Kruskal‐Wallis and Dunnʼs post‐test. ***p < 0.001, **p < 0.01

To expand analyses beyond antibody quantification, functionality determined as binding strength of Bet v 1‐specific nasal fluid IgG, SIgA and SIgM was analyzed by avidity ELISA.^5^ Whereas IgG and SIgM avidities were similar, SIgA avidities of NA were significantly higher compared to BPA (Figure 1B,C). Avidity indices represent salt concentrations when 50% of bound antibodies are eluted off the protein. This assay allows determination of individual binding strength of antibody subclasses in complex mixtures and is independent of quantities. To cope with diverse immune responses, mucous membranes comprise high‐ and low‐affinity SIgA.^1^ We thus conclude that NAs possess more high‐affinity Bet v 1‐specific SIgA compared to BPAs.

To investigate whether nasal fluid antibodies of NAs and BPAs differ in their capacity to inhibit serum IgE binding to Bet v 1, we conducted a blocking ELISA. This setup mimics allergen capture to prevent Bet v 1 binding to mast‐cell bound (mucosal) IgE. Using a serum pool of birch pollen allergics guaranteed a broad IgE repertoire and enabled testing of all nasal fluid antibodies. To relate blocking capacities to antibody isotypes, individual nasal fluids were separated into an SIgA/SIgM‐enriched and purified IgG fraction (Figure E2). Complete nasal fluid and the SIgA/SIgM fraction of NA showed significantly higher inhibitory capacities (*p* < 0.01) compared to BPA (Figure 1D). As nasal fluid SIgM levels are negligible, high‐affinity SIgA seems primarily responsible for this blocking effect.

Interestingly, complete nasal fluids of BPA lacked efficient blocking activity despite the fact that purified IgG showed some inhibitory capacity (Figure 1E). Solely in allergics, an interplay of mucosal antibody subclass interaction led to outcompeting of allergen‐IgG binding by the high abundance of low‐affinity SIgA. This bound SIgA might however in turn elute off during ELISA wash steps or, more likely, high‐affinity allergen‐specific serum IgE displaces the low‐affinity SIgA. As NAs were shown to possess more high‐affinity SIgA, such an effect was absent. Here, we first‐time demonstrate that healthy individuals exposed to birch pollen mount a distinct nasal fluid antibody repertoire toward Bet v 1. Functional high‐affinity SIgA in NA was identified as protective factor that can prevent allergic sensitization through efficient allergen capture in the nasal mucosa. Further studies will reveal the potential of SIgA as diagnostic marker to discriminate allergic and atopic individuals and to monitor the success of allergen‐specific immunotherapy.

## CONFLICT OF INTEREST

Dr. Lang reports non‐financial support from Bencard, non‐financial support from ALK‐Abelló, non‐financial support from Thermo Fisher Scientific, outside the submitted work; Dr. Ferreira reports personal fees from HAL Allergy, personal fees from Swiss Institute of Allergy and Asthma Research (SIAF), personal fees from AllergenOnline, outside the submitted work; Dr. Hawranek reports personal fees from ALK, personal fees from Novartis, personal fees from Takeda, personal fees from Bencard, personal fees from Lofarma, outside the submitted work; Dr. Gadermaier reports personal fees from Bencard, personal fees from Compare, outside the submitted work. The rest of the authors declares no conflict of interest.

## Supporting information

App S1

## References

[1] Breedveld A, van Egmond M. IgA and FcalphaRI: pathological roles and therapeutic opportunities. Front Immunol. 2019;10:553. 30984170 10.3389/fimmu.2019.00553 PMC6448004

[2] Benson M, Reinholdt J, Cardell LO. Allergen‐reactive antibodies are found in nasal fluids from patients with birch pollen‐induced intermittent allergic rhinitis, but not in healthy controls. Allergy. 2003;58(5):386‐392. 12752324 10.1034/j.1398-9995.2003.00113.x

[3] Gokkaya M, Damialis A, Nussbaumer T, et al. Defining biomarkers to predict symptoms in subjects with and without allergy under natural pollen exposure. J Allergy Clin Immunol. 2020;146(3):583. 32272131 10.1016/j.jaci.2020.02.037

[4] Dilek F, Ozkaya E, Gultepe B, Yazici M, Iraz M. Nasal fluid secretory immunoglobulin A levels in children with allergic rhinitis. Int J Pediatr Otorhi. 2016;83(4):41‐46. 10.1016/j.ijporl.2016.01.018 26968051

[5] Huber S, Lang R, Steiner M, et al. Does clinical outcome of birch pollen immunotherapy relate to induction of blocking antibodies preventing IgE from allergen binding? A pilot study monitoring responses during first year of AIT. Clin Transl Allergy. 2018;8:39. 30338052 10.1186/s13601-018-0226-7 PMC6174570

[6] Shamji MH, Kappen J, Abubakar‐Waziri H, et al. Nasal allergen‐neutralizing IgG4 antibodies block IgE‐mediated responses: novel biomarker of subcutaneous grass pollen immunotherapy. J Allergy Clin Immunol. 2019;143(3):1067‐1076. 30445057 10.1016/j.jaci.2018.09.039

