# Supplementary material for: High‐affinity Bet v 1‐specific secretory IgA antibodies in nasal fluids protect against birch pollen allergy
Source: Allergy. 2021 Mar 4;76(7):2267–70. doi: 10.1111/all.14782 (PMC8359210; doi:10.1111/all.14782)
Supplement: Supplementary file 1 — App S1 [file ALL-76-2267-s001.docx]

**Appendix S1**

**SUPPORTING MATERIALS AND METHODS**

**Study population**

For this study, we recruited 10 non-allergic (4F/6M, mean age 31.7 years) and 10 birch pollen allergic individuals (6F/4M, mean age 39.6 years) from the county of Salzburg, Austria (Table 1). Inclusion criteria for the group of non-allergics was no clinical history of birch pollen allergy and negative serum IgE ImmunoCAP towards birch and Bet v 1 (˂ 0.35 kU/l). For birch pollen allergic patients, a clinical history of moderate to severe birch pollen allergy and a positive skin prick test to birch pollen was mandatory. The patients reported no use of inhaled corticosteroids at least one week before sampling. Approval for the application of clinical data was obtained from the Ethics Commission of Land Salzburg (01/20/2011). Informed written consents were obtained from all study participants.

**Serum IgE and IgG reactivity**

In the course of the clinical testing, birch pollen-specific (t3), Bet v 1-specific (t215) and total serum IgE values were measured by ImmunoCAP (Phadia, Thermo Fisher Scientific, Uppsala, Sweden). In addition, Bet v 1-specific IgG reactivity was determined using the same diagnostic system. Details on serological and clinical data are provided in Table 1 and Table E1.

**Production of recombinant Bet v 1**

Production and characterization of recombinant Bet v 1.0101 was performed according to published protocols^1^. Briefly, using the pET28b vector system the allergen was produced in *E.* *coli* BL21 Star (DE3) as non-fusion protein and purified from the soluble fraction by standard chromatographic procedures.

**Collection of nasal fluid samples and generation of fractions**

Nasal fluid samples of 10 non-allergic individuals and 10 birch pollen allergic patients were collected directly after the birch pollen season according to a protocol of Watelet *et al*. with minor modifications ^2^. Besides allergy-related symptoms of birch pollen-allergics, study participants were free of acute or chronic nose and sinus diseases at the time of sampling. They did not receive antihistamine treatment before sample collection (with the exception of BPA2). Patients reported no use of inhaled corticosteroids at least one week before sampling, which was considered the wash out period for the respective corticosteroids. For nasal fluid collection, cotton swabs (4 per individual) were inserted one by one into the nose and turned around along the nasal mucosa for at least 2 minutes until the swabs were consistently moisturized. Afterwards, the swabs were collected in a 5 ml syringe, which was placed in a 15 ml tube. 3 ml sterile PBS was added and the syringe plunger was pushed slightly into the syringe. The remaining liquid was centrifuged into the 15 ml tube (1 min, 1000 x g, swing out rotor) and stored at -20°C until further use.

Experiments performed in this study were either accomplished with complete nasal fluids or with SIgA/IgM enriched (IgG-depleted) nasal fluid and purified nasal fluid IgG antibodies. For IgG depletion and subsequent purification, Protein G Mag Sepharose Xtra beads (GE Healthcare Bio-Sciences AB, Uppsala, Sweden) were used and the procedure was conducted according to manufacturerʼs instructions. Therefore, 100 µl beads were incubated with 300 µl nasal fluids and the mixture was incubated for 3h at room temperature on a rotary wheel. The supernatant was completely removed and IgG antibodies were eluted with 300 µl elution buffer (same volume as nasal fluid start material).

**Direct ELISA**

For direct ELISA experiments, half area microplates (Greiner Bio-One GmbH, Frickenhausen, Germany) were coated with allergen (200 ng/well protein in 25 µl PBS) overnight at 4°C. After washing with TBS, pH 7.4, 0.05% (v/v) Tween, plates were blocked with TBS, pH 7.4, 0.05% (v/v) Tween, 0.5% (w/v) BSA for 1 hour at room temperature and incubated with nasal fluids (for each patient 3 nasal fluid fractions were tested; complete nasal fluid, SIgA/SIgM enriched, and the IgG elution) overnight at 4°C. Bound allergen-specific IgE, IgG1, IgG4, IgG, SIgA as well as SIgM was detected with alkaline phosphatase-conjugated monoclonal anti-human IgE (BD Biosciences, Franklin Lakes, NJ, USA), IgG1, IgG4, IgG, IgA, or IgM antibodies (all Southern Biotech, Birmingham, AL, USA), respectively, after washing and incubation for 1 hour at room temperature. For colorimetric detection after additional washing steps 10 mM 4-nitrophenyl phosphate (Applichem GmbH, Darmstadt, Germany) was used as substrate and OD measurements were performed on a Tecan Sunrise (Tecan Group LTd., Männedorf, Switzerland) plate reader at 405/492 nm. Determination of antibody reactivities were perfomed at time points where individual subclasses have either reached the plateau phase (IgG, SIgA) or latest after 360 min (IgE, IgG1, IgG4, SIgM).

**Avidity ELISA**

For avidity ELISA experiments, half area plates (Greiner Bio-One GmbH) were coated with the allergen (200 ng/well protein in 25 µl PBS) and washed and blocked as described above. Twelve wells with 25 µl of nasal fluid sample were added on plates for each patient and incubated overnight at 4°C. On the next day, 25 µl/well sodium thiocyanate (NaSCN) (Applichem) solution in a concentration series starting from 0 M (100% value, no elution) to 3 M NaSCN (IgG, SIgM) or 6 M NaSCN (SIgA) were added and incubated for 15 min. Bound allergen-specific IgG, SIgA and SIgM was detected with AP-conjugated monoclonal antibodies and the colorimetric substrate PNPP. OD measurements were performed on a Tecan Sunrise (Tecan Group LTd., Männedorf, Switzerland) plate reader at 405/492 nm. Obtained values were background-subtracted and the percentage of bound antibody subclasses was calculated for each individual (reference point OD signal at 0M NaSCN) according to a protocol by Remarque et al.^3^. Avidity indices which represent the salt concentration at which 50% of bound antibodies are eluted off the ELISA plate were calculated from exponential trend lines (starting point 100% bound antibodies) and their corresponding formula.

**Blocking ELISA**

For blocking ELISA experiments, half area plates (Greiner Bio-One GmbH) were coated with allergen (200 ng/well protein in 25 µl PBS) and washed and blocked as indicated above. Afterwards, addition of nasal fluids (for each patient 3 nasal fluid "fractions" were tested; complete nasal fluid, SIgA/SIgM enriched nasal fluid and purified IgG elution) was performed which was followed by an overnight incubation of the plates at 4°C. After washing, a serum pool of highly birch pollen allergic patients (n=12, RAST class 5) was added in the appropriate dilution (1:40 in blocking buffer according to previous titration series with 1:2 serum dilutions starting from 1:10 - 1:1280) on the nasal fluid "fractions" as well as for the 100% control. Afterwards, the plate was incubated for 1 hour at room temperature. After additional washing steps, bound allergen-specific IgE was detected with an alkaline phosphatase-conjugated monoclonal anti-human IgE antibody (BD Biosciences) which was incubated for 1 hour at room temperature. For colorimetric detection after additional washing steps, 10 mM 4-nitrophenyl phosphate (Applichem) was used as substrate and OD measurements were performed on a Tecan Sunrise (Tecan) plate reader at 405/492 nm. Percentage of blocking was calculated from a 100% value resulting from binding of non-inhibited serum IgE to Bet v 1. Accordingly, serum IgE binding in wells incubated with nasal fluids was calculated (background-subtracted OD value in wells with nasal fluid divided by 100% signal).

**Statistical analyses**

GraphPad Prism software 7 was used for statistical analysis. Mann-Whitney test was used for comparison of study groups (non-allergic and birch pollen allergic individuals). Wilcoxon matched pairs signed rank test was used to compare the antibody reactivity in nasal fluids before and after IgG depletion. Kruskal-Wallis test with Dunnʼs post test was used to study the antibody functionality (avidity, blocking capacity) within donor groups. *P*-values < .05 were considered statistically significant. Spearman’s rank correlations of Bet v 1-specific IgE and IgG measured in serum and nasal fluid were determined for BPA patients.

**SUPPORTING REFERENCES**

1. Wallner M, Hauser M, Himly M, et al. Reshaping the Bet v 1 fold modulates T(H) polarization. *J Allergy Clin Immunol.* 2011;127(6):1571-1578 e1579.

2. Watelet JB, Gevaert P, Holtappels G, Van Cauwenberge P, Bachert C. Collection of nasal secretions for immunological analysis. *Eur Arch Otorhinolaryngol.* 2004;261(5):242-246.

3. Remarque EJ, Roestenberg M, Younis S, et al. Humoral immune responses to a single allele PfAMA1 vaccine in healthy malaria-naive adults. *PLoS One.* 2012;7(6):e38898.

**SUPPORTING FIGURES**

**Table E1. Bet v 1-specific IgE and IgG reactivity in serum and nasal fluids of NA and BA.**

|  | **Bet v 1-specific**  **serum IgE ImmunoCAP [kU/l]** | **Bet v 1-specific**  **nasal fluid IgE**  **ELISA [OD]** | **Bet v 1-specific**  **serum IgG**  **ImmunoCAP [mgA/l]** | **Bet v 1-specific**  **nasal fluid IgG**  **ELISA [OD]** |
| --- | --- | --- | --- | --- |
| NA 1 | < 0.01 | 0.034 | <2.00 | 0.507 |
| NA 2 | < 0.01 | 0.001 | 2.02 | 0.369 |
| NA 3 | < 0.01 | 0.004 | <2.00 | 0.901 |
| NA 4 | < 0.01 | 0.011 | <2.00 | 0.858 |
| NA 5 | < 0.01 | 0.008 | <2.00 | 0.900 |
| NA 6 | < 0.01 | 0.000 | <2.00 | 0.199 |
| NA 7 | < 0.01 | 0.000 | <2.00 | 0.250 |
| NA 8 | < 0.01 | 0.000 | <2.00 | 0.449 |
| NA 9 | < 0.01 | 0.000 | <2.00 | 1.464 |
| NA 10 | < 0.01 | 0.000 | <2.00 | 1.000 |
| **Mean reactivity NA** | **< 0.01** | **0.006** | **<2.00** | **0.690** |
| Correlation serum and nasal fluid reactivity NA | calculation not possible,  values below detection limit | | calculation not possible,  values below detection limit | |
| BPA 1 | 26.7 | 0.159 | 2.99 | 3.003 |
| BPA 2 | 0.7 | 0.000 | 2.97 | 2.732 |
| BPA 3 | 7.7 | 0.000 | 2.28 | 0.355 |
| BPA 4 | 16.0 | 0.000 | 2.01 | 0.291 |
| BPA 5 | 14.9 | 0.000 | <2.00 | 1.882 |
| BPA 6 | 6.2 | 0.043 | 2.62 | 2.901 |
| BPA 7 | 65.3 | 0.146 | 2.10 | 1.717 |
| BPA 8 | 4.5 | 0.045 | 5.17 | 3.005 |
| BPA 9 | 12.8 | 0.001 | 2.08 | 1.856 |
| BPA 10 | 57.0 | 0.242 | <2.00 | 2.978 |
| **Mean reactivity BPA** | **21.2** | **0.063** | **2.78** | **2.072** |
| Correlation serum and nasal fluid reactivity BPA | r_s_ = 0.524  *p* = 0.125 | | r_s_ = 0.881  *p* = 0.007 | |

NA, non-allergics; BPA, birch pollen allergics

~~

~~

**Figure E1. Serum IgE reactivity of non-allergic (NA) and birch pollen-allergic (BPA) individuals.** Total, birch pollen and Bet v 1-specific serum IgE was measured by ImmunoCAP.


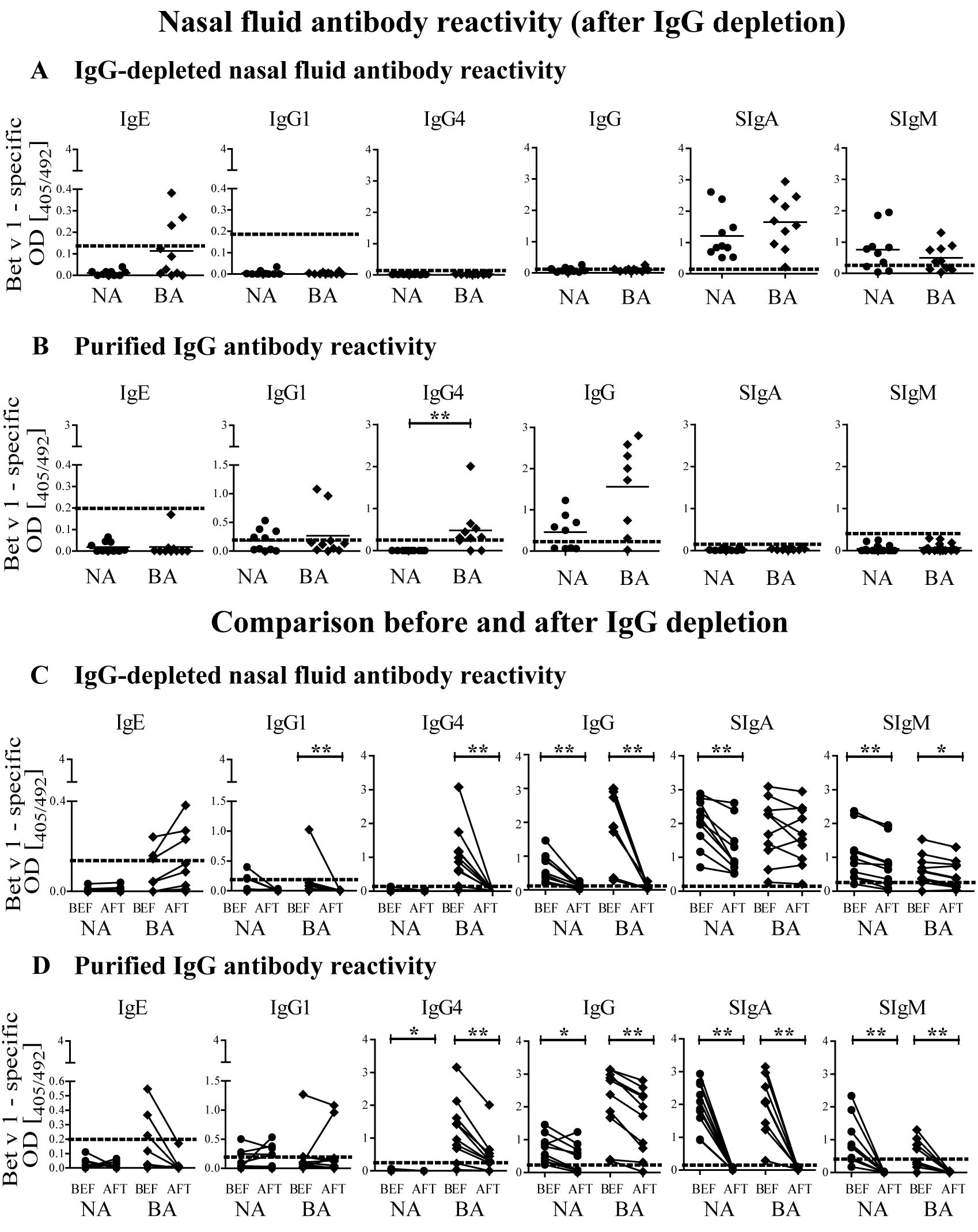


**Figure E2. Bet v 1-specific antibody reactivity of nasal fluid fractions before and after protein G purification.** Antibody subclass reactivity in A) SIgA/SIgM enriched nasal fluid fractions and B) purified IgG fractions obtained from nasal fluids of non-allergic individuals (NA) and birch pollen allergic individuals (BA). Mean levels are indicated by bars, dotted line represents the limit of detection defined as 3xSD of the antibody control. Statistical analyses were performed with Mann-Whitney test. Comparison of antibody subclass reactivity of C) SIgA/SIgM enriched nasal fluid fractions and D) purified IgG before (BEF) and after (AFT) protein G purification. OD_405/492_ of antibody subclasses was measured before and after purification by ELISA. Due to minor dilution effects during protein G purification, SIgA and IgG reactivity was found to be decreased, primarily in NAs. This indicates that the measured inhibition capacities of these two antibody subclasses could be in fact even higher. The LOD (limit of detection) was used as a cutoff value, which is calculated as three times the mean standard deviation of the blank. Wilcoxon matched−pairs signed rank test was used to compare the antibody reactivity before and after purification within study groups. **p* < 0.05, ***p* < 0.01
